# Rheological Characteristics of Soluble Cress Seed Mucilage and β-Lactoglobulin Complexes with Salts Addition: Rheological Evidence of Structural Rearrangement

**DOI:** 10.3390/gels9060485

**Published:** 2023-06-13

**Authors:** Afsaneh Taheri, Mahdi Kashaninejad, Ali Mohammad Tamaddon, Juan Du, Seid Mahdi Jafari

**Affiliations:** 1 Department of Food Process Engineering, Faculty of Food Science and Technology, Gorgan University of Agricultural Sciences and Natural Resources, Gorgan 49138-15739, Iransmjafari@gau.ac.ir (S.M.J.); 2 Food, Chemical and Biotechnology Cluster, Singapore Institute of Technology, 10 Dover Drive, Singapore 138683, Singapore; 3 Department of Pharmaceutical Nanotechnology and Center for Nanotechnology in Drug Delivery, Shiraz University of Medical Sciences, Shiraz 71348-14336, Iran

**Keywords:** β-lactoglobulin, complex coacervation, cress seed, ionic strength, rheological properties, structure

## Abstract

Functional, physicochemical, and rheological properties of protein–polysaccharide complexes are remarkably under the influence of the quality of solvent or cosolute in a food system. Here, a comprehensive description of the rheological properties and microstructural peculiarities of cress seed mucilage (CSM)-β-lactoglobulin (Blg) complexes are discussed in the presence of CaCl_2_ (2–10 mM), (CSM–Blg–Ca), and NaCl (10–100 mM) (CSM–Blg–Na). Our results on steady-flow and oscillatory measurements indicated that shear thinning properties can be fitted well by the Herschel–Bulkley model and by the formation of highly interconnected gel structures in the complexes, respectively. Analyzing the rheological and structural features simultaneously led to an understanding that formations of extra junctions and the rearrangement of the particles in the CSM–Blg–Ca could enhance elasticity and viscosity, as compared with the effect of CSM–Blg complex without salts. NaCl reduced the viscosity and dynamic rheological properties and intrinsic viscosity through the salt screening effect and dissociation of structure. Moreover, the compatibility and homogeneity of complexes were approved by dynamic rheometry based on the Cole–Cole plot supported by intrinsic viscosity and molecular parameters such as stiffness. The results outlined the importance of rheological properties as criteria for investigations that determine the strength of interaction while facilitating the fabrication of new structures in salt-containing foods that incorporate protein–polysaccharide complexes.

## 1. Introduction

Liquid–liquid phase separation or complex coacervation involves interactions between molecules that ultimately make charged polymers bind with each other, especially in protein–polysaccharide interactions [1]. Where complex coacervation occurs, solvent–polymer and polymer–polymer interactions usually bring change to the rheology of components [2]. This can be regarded as a complex formation between polymers, while finding the specifications of this formation can involve measurements of viscosity and viscoelastic properties. In particular, the said properties can be detected in the isolate–beet pectin of ovalbumin/dextran sulfate [3], pea protein [4], and rice bran protein–flaxseed gum [5]. In other words, despite the macromolecular importance of the interaction of polymers (i.e., type and intensity of chemical interaction, structure, and conformations), the rheological properties of the complex can remarkably affect both macromolecular perspective and application of complexes.

With its small size, β-lactoglobulin (Blg) is a compact, dominant, globular part of whey protein, and has received considerable attention due to its extensive functional properties (i.e., foaming, emulsification thickening, delivery of lipophilic micronutrients, and bioactive molecules) [6]. Due to electrostatic repulsions between its monomers, Blg solutions are stable. Near the isoelectric point (pI = 5.1), it, however, starts to aggregates and form a different structure depending on the ionic strength and pH of the solution [7]. There is a possibility to impress the aggregation, thereby generating functional properties of Blg when it enters complexation with other biopolymers such as acacia gum [8], pectin [9], and κ-carrageenan [10].

Cress seed mucilage (CSM) is mostly comprised of carbohydrates (85%) and is extracted from *Lepidium sativum.* The molecular weight of CSM is approximately 540 kDa. Its zeta potential is −10.78 ± 0.19 mV and it is characterized by an intrinsic viscosity of 13.3 dL/g. While it is regarded as a polyelectrolyte molecule, CSM comprises a rigid chain conformation and contains D-galacturonic acid and D-glucuronic acid [11]. As a novel source of hydrocolloids, CSM is indicated by unique functional properties and physicochemical features in the scientific literature [12,13]. It can withstand a diversity of pH values, ionic effects of NaCl and CaCl_2_, and heat effects by thermal processing. These properties make CSM a suitable option in formulating foods.

The polyelectrolytic and anionic features of this hydrocolloid can facilitate the formation of mucilage-based complexes with protein molecules, while also having the ability to participate in electrostatic reactions. In previous research, the electrostatic interactions of cress mucilage with Blg have been studied and approved. While electrostatic attraction has a significant role in regulating the integrity of Blg with its polar charges, the attraction is specifically sourced from cationic amino groups and from those on CSM macromolecules (i.e., anion groups). Thus, the ionic strength of the medium can largely suppress interactions while causing complex formations in insoluble/soluble forms and changing the properties of physicochemical features. On the other hand, the electrostatic interaction between CSM and Blg can be affected by modifying the solvent through the use of additives such as salts. Accordingly, the compatibility (i.e., miscibility of polymers with other polymers) of the ingredients, hydration parameters (coil radius and volume), hydrodynamic parameters (chain flexibility; stiffness), and their conformation can be altered by changes occurring in the solvent, which is assessable by rheological measurements [14].

In multiphase systems such as polymer blends, there is a possibility to monitor the structure complexation with the investigation of flow behavior and especially with viscoelasticity studies. Therefore, any changes in the morphology of the CSM–Blg structure were assessed at different salt conditions to derive a relationship between the structural variations and rheological characteristics. In this context, the present paper addresses the multifaceted dimensions of rheological features (i.e., flow behavior, formation of complexes and interactions) along with the aggregation behavior and structural changes of the CSM–Blg complex in the presence of NaCl and CaCl_2_. The results of the current research can shed light on specific potentials for prospective uses in pharmaceutical media and in functional foods that contain salt. These can benefit from the CSM–Blg complex in their stability and preservation.

## 2. Results and Discussion

### 2.1. Morphological Analysis and Steady Shear

Exploring the suitability of models was aimed at achieving precise indications of flow behavior, which involved 2 *w*/*v*% CSM–Blg solutions. Rheological models that were time-independent in this research were used in measuring data of shear stress–shear rate in CSM–Blg solutions. Despite that these models indicated large values of R^2^, the experimental data suggested that the Herschel–Bulkley model performed optimally (i.e., with the highest R^2^) (Table 1). Regarding the flow behavior of CSM, the Herschel model has already been described in the available literature as a suitable option for this purpose [15]. Considering the higher coefficient of determination in association with the CSM–Blg complex in this model, polysaccharides are important in directing the flow properties of the protein–polysaccharide complex. A shear thinning (pseudoplastic) behavior (*n_H_* < 1) was observed in CSM–Blg complexes, which partly emanated from differences in salt concentrations, alignment of anisotropic particles, interacting particles, and their rearrangements, and an applied change in shear rate (0.1–100 s^−1^) in combination with the structural relaxation of complexes [16,17]. The highest *n_H_* and the lowest *k* were attributable to the CSM–Blg, meaning that the salt incorporation increased the pseudoplasticity (n) and thickening (k) properties of the complex. In general, adding the electrolyte to polymer solutions stabilized by an extending electrical double layer (EDL) results in the compression of the layer (Debye–Hückel theory), and addition of further electrolyte led to the collapse of the double layer, loss of structure, and a corresponding shear thinning behavior [18,19].

**Figure 1:**
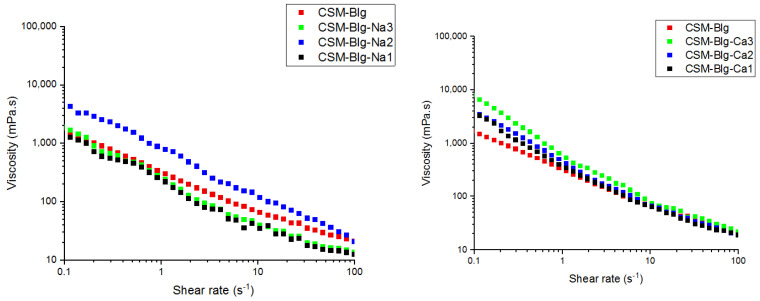
CSM–Blg complexes and their viscosity values, as affected by different salt concentrations.

The strength of attraction-forced electrostatic interactions between protein–polysaccharides can be monitored in association with viscosity. Due to the higher viscosity of polysaccharides in comparison with proteins, the increase in protein viscosity after complex formation contributed to the formation of continuous network structure through polymers that take part in complex coacervation [20,21]. In polyelectrolyte molecules such as CSM, viscosity is affected by ionic strength. In CSM–Blg complexes, the highest value of apparent viscosity was measured in the treatment group that had 10 mM CaCl_2_ (7.95 Pa.s at 0.1 s^−1^) and 50 mM NaCl (4.97 Pa.s at 0.1 s^−1^) (Figure 1), which can be due to a tightly packed structure that generated the strongest electrostatic interactions of attraction between CSM and Blg molecules [22,23]. Hasanvand and Rafe (2018) conducted research on the complexation of rice bran protein–flaxseed (*Linum usitatissimum* L.) and showed that the complex had higher levels of apparent viscosity due to its denser structure and higher electrostatic attraction between the gum and protein molecules. Liu et al. (2017) reported that enhancement of viscosity can be attributed to a higher degree of compatibility among biopolymers at the medium levels of salt which increased the prevalence of biopolymers in the continuous phase, thereby making entanglement more probable in the chains [24].

**Figure 2:**
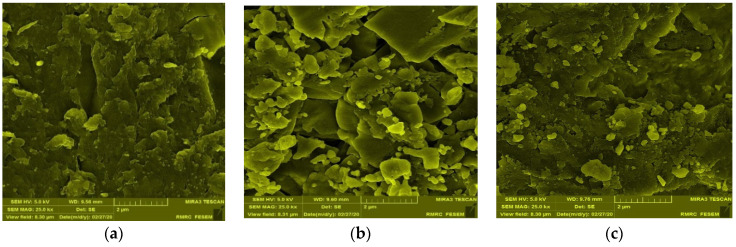

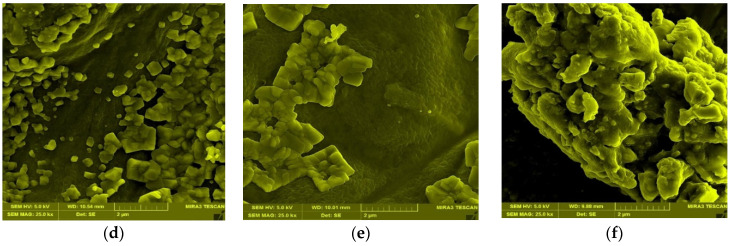
FE-SEM images of CSM–Blg complex samples. (**a**) Table 1, sample CSM–Blg–Na_1_; (**b**) Table 1, sample CSM–Blg–Na_2_; (**c**) Table 1, sample CSM–Blg–Na_3_; (**d**) Table 1, sample CSM–Blg–Ca_1_; (**e**) Table 1, sample CSM–Blg–Ca_2_; and (**f**) Table 1, sample CSM–Blg–Ca_3_. All images were taken through similar magnification, with 2 μm as the scale bar.

The higher the NaCl concentration, the lower the viscosity of the CSM–Blg complex system. This trend can be assigned to the reduced charge density of counter-ions with the polymer molecules which, in turn, declined the electrostatic attraction of the charged groups on the polymer chains [25]. An investigation into the aggregation behavior of CSM–Blg complexes with FE-SEM (Figure 2a–c) showed that NaCl, at low concentrations in a biopolymer mixture, ultimately led to structures that were tightly packed. Nonetheless, adding more salt beyond this point (50 mM) caused a dissociation of CSM–Blg agglomerates (i.e., more loosely packed structure) due to the screening of charges by dissolved Na^+^ and Cl^−^. Similar findings were reported by Zhang et al. (2021), who examined the rheological characteristics of the pea protein isolate–chitosan complex. Accordingly, it was reported that the maximum amount of packed network structure of the complex was prepared at 0.1 M NaCl, whereas higher concentrations caused a loosening of the complex’s structure [25,26].

FE-SEM images showed how the biopolymer mixture contained CaCl_2_ at small concentrations (2 and 5 mM), implying that the structures are more packed, although further increases in the CaCl_2_ concentration culminated in a slightly porous structure (Figure 2d–f). An increase in the apparent viscosity was reported as an increase in porosity at high ionic strength (CaCl_2_) in the chitosan–whey protein isolate complex [27,28]. In this regard, W. Wang et al. (2018) studied the *Mesona blumes* polysaccharide–soy protein complex and showed rougher and larger networks, surfaces, and pores when using high CaCl_2_ concentrations (0.015–0.02 M), compared to low concentrations (0.005–0.01 M). In sum, the CSM–Blg complex had a viscosity value that showed that the apparent viscosity was increased by the action of CaCl_2_ at high concentrations. This happened through the porosity of the structure, whereas quite the opposite occurred when using NaCl [29].

As a general rule of thumb, for biopolymer complexes to act as structuring agents in food formulation systems, they must demonstrate shear thinning characteristics and high viscosity. Moreover, the viscoelasticity (elastic component predominates over the viscous component) has the same significance.

### 2.2. Frequency Sweep Results

**Figure 3:**
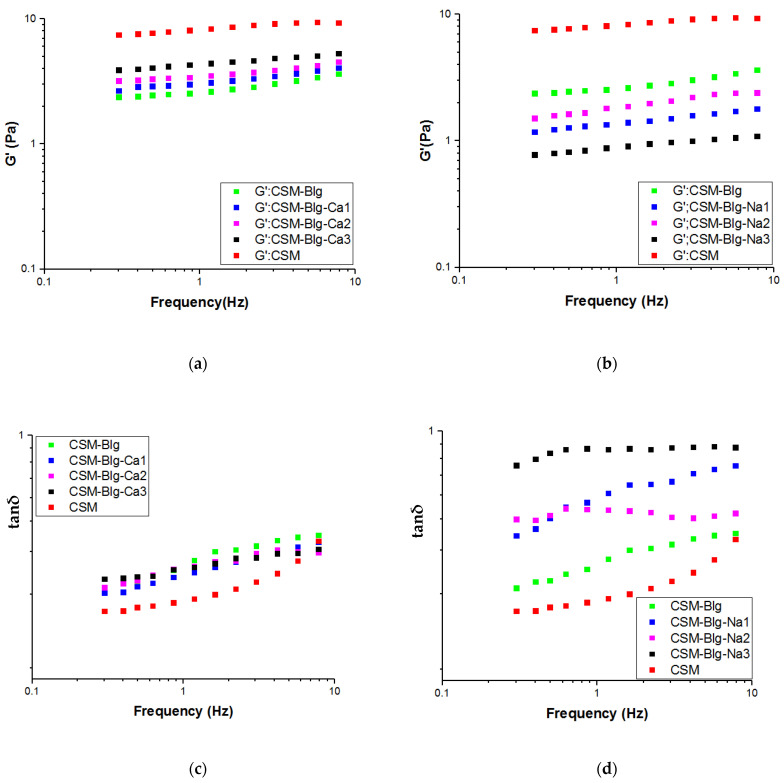
(**a**,**b**) Variation of *G*′ of CSM–Blg (frequency range of 0.1–10 (Hz) at 25 °C) with different concentrations of CaCl_2_ and NaCl, respectively; (**c**,**d**) variation of tan *δ* of CSM–Blg with different concentrations of CaCl_2_ and NaCl, respectively.

In polymer blends (as a multiphase systems), the strength of complexation and structure of the biopolymers can be monitored by studying the viscoelasticity. For the evolution and to reach the ideal from the structure, it is necessary to applied small strain in experimental condition, so the dynamic oscillatory method may obtain the true responses. Here, Figure 3a–d illustrate the tanδ in CSM–Blg complexes and depicts how G’, G” values are frequency-dependent. These details are required for an accurate understanding of the different effects caused by calcium concentrations (2–10 mM) and sodium ion concentrations (10–100 mM). In comparison with Blg–CSM complexes and in the same concentration, the CSM had a bigger G′ and G″ values. A single-protein biopolymer, especially globular protein (Blg), was unable to create viscoelastic properties at low concentrations. Thus, after its interaction with polysaccharides, the viscoelastic properties of the Blg–CSM complex probably decreases, compared to the polysaccharide alone [30].

Higher values of G*′* were compared to the low values of *G″* in the analyses of frequency, which demonstrates that a network structure of an interwoven gel was created by all CSM–Blg complexes. In fact, this confirms the interactions between Blg molecules (weak elastic property) and CSM and highlights the role of CSM in terms of rheological features. Similar gel features that mimic frequency dependence (*G′*, *G″*) have been indicated in previous research on soy protein isolate–chitosan as a matrix for protein–polysaccharide complexes [31]. Other matrixes in the available literature include pea protein isolate–sugar beet pectin [32] and gelatin–gum Arabic [33]. Nonetheless, where biopolymer systems comprised ovalbumin–gum Arabic as a matrix for complexes, a viscous behavior became prominently apparent [34].

Adding sodium ions lowered the modulus further, whereas calcium ions induced an increase in the modulus but did not recover the CSM solution modulus. In comparison with CaCl_2_ and NaCl, where *G′* varied in response to CaCl_2_, *G′* values increased in response to higher concentrations of CaCl_2_ (Figure 3a). While greater values of *G′* reflect a network structure with stronger internal connection and construct, measurements of viscoelasticity showed that adding CaCl_2_ acted in favor of the interaction between CSM and Blg. The viscoelastic properties of CSM–Blg complex with the addition of NaCl were inferior to that of the complex in the presence of CaCl_2_. In response to higher NaCl concentrations (10–100 mM), both dynamic *G′* and *G″* decreased in value and reached their lowest in response to NaCl = 100 mM, suggesting how weaker gel network structures gradually grow deformed in the presence of NaCl. On the other hand, using 50 mM NaCl caused an increase in the value of *G′*, although a salient difference still occurred in comparison with the salt-free sample (Figure 3b). According to the literature, Xiong et al. (2016) studied the complexation of ovalbumin (OVA)–chitosan (CS) and reported that a stronger network structure between OVA and CS led to a high value of *G′* in response to 50 mM NaCl [34]. Meanwhile, Xiong et al. (2017) reported that a high range of ionic strength (20–400 mM) in an ovalbumin–carboxymethylcellulose complex was the precursor of a decrease in the viscoelastic properties of the complex through shielded biopolymer charges, thereby making a loosely compacted structure [35].

The phase angle (tan *δ*) is another important numerical value that can facilitate a precise evaluation of a given complex in terms of viscoelastic behavior. In fact, the ratio of *G″/G′* serves as a definition of the phase angle [36,37]. As can be seen in Figure 3c,d, where *G″*/*G′* < 1, solid elasticity became apparent in the CSM–Blg complexes. Accordingly, a strong solid elasticity was observed in the CSM–Blg complexes when CaCl_2_ was used. The tanδ value grew small in response to low CaCl_2_ concentrations (2 mM), compared to high concentrations. In contrast, tanδ became small in response to low NaCl concentrations (10–50 mM), but increased in response to the high NaCl concentration (100 mM) [38]. This finding was similar to previous results by W. Wang et al. (2018) regarding complexation in *Mesona blumes* polysaccharide–soy protein isolates, demonstrating that high sodium ion concentrations (0.02 M) can result in poorer solid elasticity in a complex. Generally, the findings showed that the frequency dependence of *G′*, *G″,* and tan *δ* is sophisticated and considerable in the CSM–Blg system, although it is heavily dependent on the salt concentration.

To assess (*n′* and *n″*) and (*k′* and *k″*) from Equations (5) and (6), nonlinear regression analysis (power law models) were taken from oscillatory measurements and applied onto all complexes. As observed, the model can finely estimate both *G′* and *G″* alterations vs. frequency (R^2^ = 0.95–0.99) and (R^2^ = 0.94–0.99), respectively. Since the *kʹ* value was much higher than the *k″* value in the case of all complexes (*k″*/*k′* < 1, Table 2), the previous results can be confirmed regarding the gel behavior of these systems [39]. Incorporating the CaCl_2_ into CSM–Blg complexes caused a decrease in *n′* and *n″* values, whereas significant increases were observed in the magnitudes of *k′* and *k″*, thereby manifesting a decrease in frequency dependence and a stronger gel behavior.

The values of (*n′* and *n″*) and (*k*’ and *k″*) are indicative of the number and strength of interactions, respectively. If all of the indexes (*n′*, *n″*, *k′,* and *k″*) were enhanced, the elastic part is dominated, which means the solid viscoelastic-like response is more prominent in the interaction of polymers. In contrast, a decline in these indexes reflects the dominance of the liquidlike viscoelastic features and the higher fluidlike behavior in the interactions [40]. As demonstrated in Table 2, the magnitudes of *n′* and *n″* for CSM–Blg–Na ranged between (0.105–0.148) and (0.137–0.311), indicating the dominance of liquidlike character with the addition of NaCl (100 mM).

**Figure 4:**
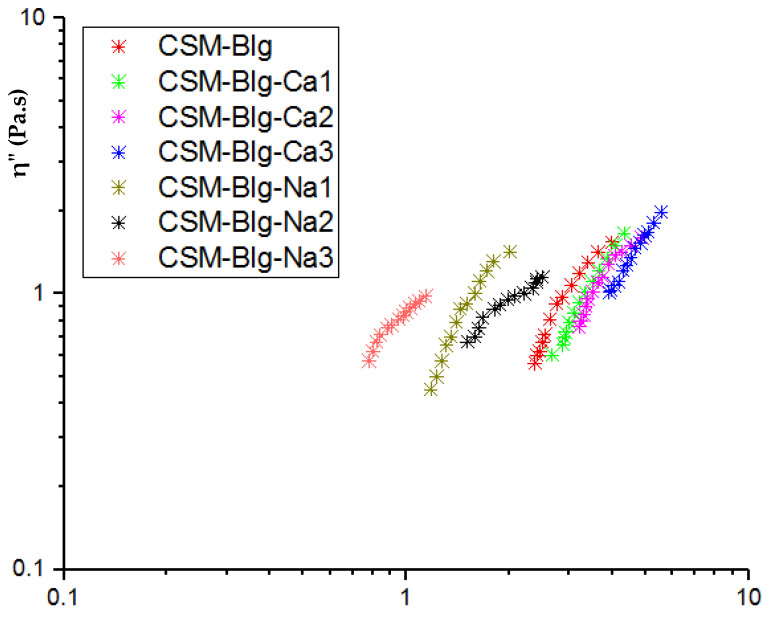
Cole–Cole plot of the imaginary part of viscosity (*η″*) versus dynamic viscosity *η′* for CSM–Blg complexes for various salt concentrations.

The Cole–Cole graph demonstrates the relaxation and miscibility of the polymer blends. This graph can be also used to identify the structural changes [41]. The Cole–Cole plot indicates the frequency dependence of the imaginary viscosity *η″ (G′*/*ω*) versus the real viscosity *η′* (*G″*/*ω*) (Figure 4). The curves did not superimpose with each other in the presence of sodium and a high concentration of calcium, showing that the complex creates various structures after the addition of salts. Moreover, the Cole–Cole plot of all CSM–Blg complexes showed a semicircular shape with one arc corresponding to the compatibility and homogeneity of complexes at the presence of salts [42]. At high concentrations, the plots showed a clear deviation from the semicircular shape and indicated a linear variation of the storage viscosity versus the loss viscosity. This represents a gel structure and demonstrates the occurrence of maximum particle–particle interactions at high calcium ion levels. In an ideal *Maxwellian fluid*, the semicircular and smooth curves suggest the proper miscibility of the polymer. Any deviation from this shape demonstrates the incompatibility of participant polymer in complexation. The power law model was used for the evolution of the deviation from the semicircular shape. The power law index (*α*) is indicated in Table 2. CSM–Blg demonstrated the worst miscibility at the presence of low and high concentrations of NaCl.

### 2.3. Dilute Solution Properties

The intrinsic viscosity (*η*) is a convenient way to measure the hydrodynamic volume occupied by individual macromolecule coil and is closely related to the size and topological structure of the macromolecular chains [43]. The results of fitting the intrinsic models are demonstrated in Table 3, which suggests high accuracy for Higiro 1 models (R^2^ = 0.93 to 0.98) for all CSM–Blg complexes, showing the efficiency of these models to measure the intrinsic viscosity of CSM–Blg complexes in various solvents/cosolutes (Table 3). As indicated in Table 3, the intrinsic viscosity was remarkably affected by enhancing the ionic strength (I). The intrinsic viscosity of CSM–Blg in deionized water was reported to be 0.46 ± 0.03 dL/g, which is higher than those of other complexes in presence of salts (see Table 3). This confirms the reduced quality of solvent and synergy behavior of [*η*] between Blg and CSM with cosolute (NaCl and CaCl_2_) [44]. In the case of Na^+^ ion, an increase in I from 0.01 to 0.1 M resulted in a drop in CSM–Blg intrinsic viscosity compared to Ca^+2^ from (0.46 to 0.18 dL/g) and (0.46 to 0.28 dL/g), respectively. Such a decrease with the addition of NaCl can be assigned to the enhanced charge screening of polymer chain inducing more contracted conformation and causing a reduction in the hydrodynamic size of the molecule [45]. With the increase of CaCl_2_ in dispersions, intrinsic viscosities decreased (from 0.46 to 0.32 dL/g and from 0.46 to 0.25 dL/g) in the presence of 2 and 5 mM CaCl_2_ (Table 3), respectively. However, a slight increase was detected at high calcium concentrations.

**Figure 5:**
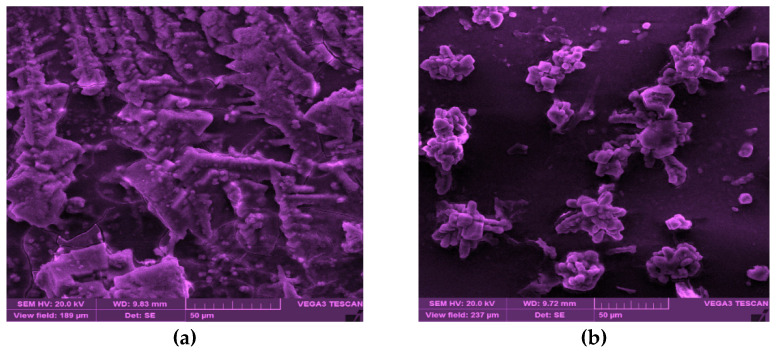
The SEM images of CSM–Blg complexes: (**a**) without CaCl_2_ and (**b**) with 10 mM CaCl_2_ (scale bar: 50 μm).

For a deeper insight into the effect of the high concentration of calcium, SEM image analysis was carried out (see Figure 5). SEM images of the complex with no CaCl_2_ showed that most of the particles are clustered together, forming a rod shape. These big microparticles are mainly composed of small nanoparticles in various size ranges (roughly from 50 nm to several micrometers). Figure 5 depicts the complex with CaCl_2_ which exhibited a certain change in morphology with an organized structure. Some particles are tightly combined, and nanoparticles and microparticles showed a tendency to form an organized structure. i.e., hexagonal-like. In other words, the rearrangement of the particles to new structures could result in the variation of the rheology of this particle at high concentrations.

**Figure 6:**
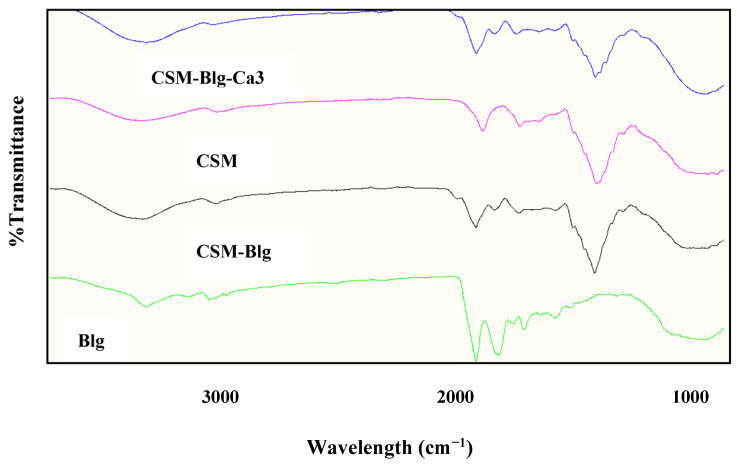
FTIR spectra of biopolymers (CSM, Blg) and complexes (CSM–Blg and CSM–Blg–Ca_3_) prepared at pH, CSM–Blg mixing ratio, CSM concentration, and Blg concentration of 3.5, 1:2, 0.35% *w*/*v,* and 0.1% *w*/*v*, respectively.

FTIR spectroscopy is a sensitive diagnostic tool for monitoring conformational alterations in biopolymers. Considering Blg, the observed peaks correspond to the β-sheet, random coil, α-helix, β-turn, and β-antiparallel sheet at 1613–1637 cm^−1^, 1637–1644.5 cm^−1^, 1644.5–1662 cm^−1^, 1662.5–1682 cm^−1^, and 1682–1689 cm^−1^, respectively. These parameters were previously confirmed by [46]. In our study, the strongest peak emerged at 1629 cm^−1^ for Blg. This peak describes how the native protein is organized in solution by the β-sheet. After the formation of the nanocomplex, a shift can be observed to 1630 cm^−1^ and 1628 cm^−1^ in Blg–CSM and Blg–CSM–Ca_3_, respectively, highlighting the possibility that Blg preserved its structures, suggesting the role of CSM in the reorganization of structure. A relatively big surface area exists in the β-sheet structure and can benefit the formation of ordered hydrogen bonds [47]. Thus, it can be inferred that hydrogen bonds contributed to the creation of CSM–Blg complexes. With a progressive amorphous state, the α-helix protein structure converts to the β-sheet structure when the solution comprises polysaccharides (Souza and Rojas (2016)). Sankalia et al. (2007) demonstrated that when biopolymers are mixed, they are thermodynamically stable. Thus, intermolecular interactions dominate the FTIR spectra of the mixed polymers, showing a deviation from their constituent polymers. In contrast, FTIR spectra overlapped with the mixture of two incompatible polymers (phase-separated). The FTIR results indicated that these two polymers are thermodynamically compatible (Figure 6). The thermodynamic compatibility of protein–polysaccharide complexes by FTIR was also reported by Raie et al. (2018) in a study on high methoxy pectin–whey protein and β-lactoglobulin and basil seed gum [48].

In sum, wherever the same ionic strength applied, the divalent ion (Ca^2+^) had a more prominent effect on intrinsic viscosity of CSM–Blg, compared to the monovalent type (Na^+^). A possible reason for this is the occurrence of molecular crosslinking among CSM and calcium ions, as well as various degrees of aggregation which lead to a diverse extent of molecular contraction [49]. The alteration of coil radius (*R_coil_*) and its corresponding volume (*V_coil_*) in CSM–Blg complexes was strongly affected by ion type and concentration, as demonstrated in Table 3. *R_coil_* values of CSM–Blg complexes were higher and were reduced with an increase in the salt level, which could be ascribed to less-extended CSM chains and more compact structure. The changes of intrinsic viscosity, coil radius, and volume are interdependent. These findings are corroborated by changes in the intrinsic viscosity of CSM–Blg samples. As an indicator of polymer chain stiffness, salt tolerance correlates with a higher degree of chain flexibility. A higher level of dependency on ionic strength in intrinsic viscosity is usually accompanied by a greater salt tolerance. Based on Equation (17), the plot of the intrinsic viscosity was obtained vs. the reciprocal of the square root of ionic strength (I^−0.5^) for evaluation of the CSM–Blg stiffness parameter in the presence of Na^+^ and Ca^2+^ ions. As seen, the stiffness parameter was lower in the presence of the monovalent ion NaCl (3.34) as compared to the divalent one (CaCl_2_) (14.46), demonstrating a stiffer backbone of CSM–Blg in the presence of divalent ions. In other words, contraction caused by Ca^2+^ in CSM–Blg complex was greater than that of Na^+^, which is consistent with the results of viscosity and SEM images.

## 3. Conclusions

The high values of *G′* compared to *G″* in the frequency ranges were demonstrated by oscillatory measurements, indicating network structures with strong interconnections in their gel formations of the complexes. In part, the said observation can be explained by the fact that Blg and CSM molecules have electrostatic interactions between themselves, and this can occur even when salt is present in the medium. It was observed that solvent quality decreased with increasing the salt concentration, which can be ascribed to the larger reduction of the intrinsic viscosity and miscibility through monovalent cations compared with the divalent cations. The system was homogenous and compatible as approved by dynamic rheometry based on Cole–Cole plot. Since the rheological properties of the high concentrations of calcium were different from the other samples, the analysis of SEM indicated the rearrangement of the CSM–Blg complex in the presence of 10 Mm CaCl_2_. The rheological features demonstrated how important these concepts are in understanding the mesoscale structures, while validating previous standings on rheological data through evaluations of interactions that occur in complex polymers. More research can actualize potential applications of such relevance in foods, especially by considering salt treatments.

## 4. Materials and Methods

### 4.1. Materials

Cress seeds were locally obtained from a reputable seed seller. CaCl_2_ (anhydrous), Blg (with 93% purity), sodium hydroxide (NaOH), and hydrochloric acid (HCl) were sourced from Sigma-Aldrich (Schnelldorf, Germany). Deionized water was used for diluting the solutions when necessary (Nanopure Infinity, Barnstead International, Dubuque, IA, USA).

### 4.2. Cress Seed Mucilage Extraction and Purification

Extraction and purification processes of cress seed mucilage were carried out appropriately [50]. Briefly, water–seed ratio was 30:1, with an alkaline pH value (10) and 15 min of soaking, which rendered extraction conditions optimum. After extracting the slurry, ethanol precipitation was applied for the purification of mucilage. This was followed by stirring the mucilage with ethanol for 10 min (1:3 ratio, mucilage volume: ethanol volume). Stirring was followed by centrifuging the mixture at 15,000 rpm for 3 min at 25 °C. Then, freeze-drying was applied as a treatment on the mucilage to make it dehydrated (LD Plus Alpha, Martin Christ, Germany). The chemical composition of the powdered CSM comprised 12.85 ± 0.63% moisture, 0.91 ± 0.02% pr1otein, 7.93 ± 0.03% ash and 81.7 ± 0.8% total carbohydrate [50].

### 4.3. Preparation of CSM–Blg Complexes

To prepare stock solutions of Blg and CSM, the two components were dissolved in deionized water to reach 20 mg/mL and 30 mg/mL, respectively. Then, by post-blending acidification, CSM–Blg complexes were created. CSM was added to Blg dropwise. The variables were selected according to previous methods [50]. For the preparation of CSM–Blg–Ca and Na complexes, CaCl_2_ and NaCl were added to the aforementioned mixture, and the resultant dispersion was mildly stirred for 2 h. The final contents of CaCl_2_ and NaCl were in the range of 2–10 mM and 10–100 mM, respectively. Salt concentrations were set out according to the stability of CSM in the said range of salt content [51]. Ultimately, the procedure involved freeze-drying the nanocomplexes.

### 4.4. Rheology

#### 4.4.1. Steady Shear Values

Within 0.1–100 1/s, the various properties of steady shear flow (CSM–Blg) (20 °C) were determined by a rheometer (MCR 301, Anton Paar, GmbH, Graz, Austria). After applying shear thinning models, the experimental curves that describe the flow behavior were fitted by the Ostwald–de Waele (or power law), Herschel–Bulkley model, Bingham, and Casson (Equations (1)–(4)):

(1) $$\tau =k_{p}{\dot{\gamma }}^{n}$$

where *n* and *k_p_* describe the flow behavior index (dimensionless) and power-law consistency coefficient (Pa·s^n^), respectively.

(2) $$\tau =\tau_{0H}+k_{H}{\dot{\gamma }}^{n_{H}}$$

where *n_H_* describes flow behavior index (dimensionless), *k_H_* defines the consistency coefficient (Pa·s^n^), and *τ*_0*H*_ is the yield stress (Pa) according to the Herschel–Bulkley model.

(3) $$\tau =\eta_{B}\dot{\gamma }+\tau_{0B}$$

where *τ*_0*B*_ and *η_B_* are the Bingham yield stress (Pa) and Bingham plastic viscosity (Pa s), respectively.

(4) $$\tau^{0.5}=\tau_{C}^{0.5}+\eta_{C}\left({\dot{\gamma }}^{0.5}\right)$$

where *τ*_0*C*_ and *η_C_* are the Casson yield stress (Pa) and Casson plastic viscosity (Pa s), respectively.

The flow behavior of nanocomplex solutions was depicted by a best-fitting model which had the highest coefficient of determination (R^2^).

#### 4.4.2. Oscillatory Measurements

The CSM–Blg had its rheological behavior of dynamic shear properties determined through a finite level of total biopolymer concentration (2% *w*/*v*) at three levels of concentration for NaCl (10, 50, 100 mM) and CaCl_2_ (2, 5, 10 mM) via a rheometer (MCR 301, Anton Paar, GmbH, Graz, Austria). Before measuring the dynamic shear, each sample was maintained for 10 min in storage at 25 °C. The linear–viscoelastic region (LVR) was estimated by strain sweep determinations before frequency sweep tests, where *G″* and *G′* performed without dependency on strain amplitude. In this context, within 0.01–100% of strain, a constant frequency of 1.0 Hz enabled the strain sweep determination (25 °C). While the LVR circumstances applied, frequency sweep tests ran at a constant strain amplitude (0.1%). In the LVR region, 0.01–10 Hz frequencies were considered for frequency sweep determinations. Frequency dependence of the storage modulus (*G′*) and loss modulus (*G″*) was examined accordingly through the power law model [52]:

(5) $$G^{\prime }=k^{\prime }\;\omega^{n\prime }$$

(6) $$G\prime \prime =k\prime \prime \omega^{n\prime \prime }$$

where *k′* and *k″* (Pa·s^n^) are constants; n′ and n″ are the viscoelastic components of elastic and viscous moduli, and ω denotes the oscillatory frequency (Hz).

The miscibility of different semidilute CSM–Blg complexes (2% *w*/*w* total concentration) at different salt levels was investigated by Cole–Cole plots. To this end, the imaginary (*η"*) part of the viscosity was plotted against its real (*η′*) component [53].

(7) $$\eta \prime \prime =\frac{G\prime }{\omega }\;$$

(8) $$\eta \prime =\frac{G\prime \prime }{\omega }$$

#### 4.4.3. Intrinsic Viscosity

A thermostatic water bath was used for hosting a U-tube capillary viscometer (Cannon Instruments Co., State College, PA, USA) in suspension form. The conditions were under an accurate temperature control (25 ± 0.1 °C), by which relative viscosity (*η_rel_*) was measured along with specific viscosity (*η_sp_*) as follows (Equations (9) and (10)):

(9) $$\eta_{rel}=\frac{\eta }{\eta_{s}}=\frac{t}{t_{0}}$$

(10) $$\eta_{sp}=\eta_{rel}-1$$

where *t* and *t*_0_ are the flow times of sample and pure solvent (deionized water), respectively. The intrinsic viscosity (*η*) was calculated by the models below [54]:

(11) $$\mathrm{Huggins}\;\mathrm{equation}:\;\frac{\eta_{sp}}{C}=\left[\eta \right]+K\prime {\left[\eta \right]}^{2}C$$

(12) $$\mathrm{Kraemer}’s\;\mathrm{equation}:\;\frac{ln\eta_{rel}}{C}=\left[\eta \right]+K\prime \prime {\left[\eta \right]}^{2}C$$

(13) $$\mathrm{Tan}\mathrm{glertpaibul}\;\mathrm{and}\;\mathrm{Rao}\;\mathrm{equation}:\;\eta_{rel}=1+\left[\eta \right]C$$

(14) $$\mathrm{Higiro}\;\mathrm{et}\;\mathrm{al}.\;\mathrm{equations}:\;\eta_{rel}=e^{C\left[\eta \right]}$$

where c is the solution concentration, and *K′* and *K″* represent the Huggins and Kraemer constants, respectively.

#### 4.4.4. Hydrodynamic Coil and Volume Radius, Stiffness Parameter

Hydrodynamic coil radius (*R_coil_*) and hydrodynamic coil volume (*V_Coil_*) were estimated by the following equations (Equations (15) and (16)) [55]:

(15) Rcoil=[3[η].Mw10π.NA]13

where *N_A_* is Avogadro’s number and *M_W_* (kDa) denotes weight–average molecular weight.

(16) $$V_{coil}=\frac{4}{3}\pi R_{coil}^{3}$$

The ionic strength dependence of the intrinsic viscosity of polyelectrolytes usually follows the empirical Smidsrød and Haug (1971) equation which allows for describing the macromolecular chain stiffness depending on molecular weight:

(17) $$\left[\eta \right]=\left[\eta_{\infty }\right]+SI^{-0.5}$$

where $\eta_{\infty }$ is the intrinsic viscosity at infinite ionic strength and corresponds to conformations unaffected by electrostatic interaction, and *S* (also known as salt tolerance) represents a comparative criterion for evaluation stiffness macromolecular chains [56].

### 4.5. Morphological Observation

To arrive at a sufficient description of variations in nanocomplexes within the specifications of salts and their features, the procedure involved placing samples on an FE-SEM stub for field-emission scanning electron microscopy. After coating each sample with an Au–Pt layer, electron conductivity was supposedly exposed to the surface of the sample. High-pressure vacuum (10^−5^–10^−6^ Pa) was used for taking the images (5–10 kV). The structural changes were also examined by scanning electron microscopy (SEM, TESCAN-Vega 3, Czech). A drop of the suspension was dripped onto glass stubs and placed on the surface of the sample stub, which was then left to dry. The stub was then coated with a thin (<20 nm) conductive gold layer using a sputter coater (Quorum Technologies, Lewes, UK) before imaging. After preparation, the morphological features of each sample were visualized by electron microscopy.

### 4.6. Fourier-Transform Infrared (FTIR) Spectroscopy

Chemical structures of Blg, CSM, CSM–Blg, and CSM–Blg–Ca were described with the assistance of a Tensor-II FTIR spectrometer (Bruker Co., Bremen, Germany) at 400–4000 cm^−1^ wavelength range.

### 4.7. Statistical Methods

In triplicate, the measurements were deemed reliable and with few errors. Mean values ± standard deviation were considered to present experimental data. The Origin Pro 9.1 software (Origin Lab Co., Northampton, MA, USA) was used for generating figures. Differences between means were determined by one-way analysis of variance (ANOVA). Duncan’s comparison validated the analysis of variance (*p* < 0.05) via SPSS 23.0 (IBM^®^ Armonk, NY, USA).

## Figures and Tables

**Table 1:** Steady shear rheological parameters of different CSM–Blg complexes at 25 °C.

Model		CaCl_2_ (mM)				NaCl (mM)		
	0	2	5	10	0	10	50	100
	CSM–Blg	CSM–Blg–Ca_1_	CSM–Blg–Ca_2_	CSM–Blg–Ca_3_	CSM–Blg	CSM–Blg–Na_1_	CSM–Blg–Na_2_	CSM–Blg–Na_3_
Power law								
kp (Pa·sn)	3.48 ± 0.126 d	4.5 ± 0.084 c	4.92 ± 0.133 b	7.15 ± 0.152 a	3.48 ± 0.126 b	2.37 ± 0.110 d	7.02 ± 0.148 a	2.70 ± 0.110 c
np	0.326 ± 0.007 a	0.214 ± 0.002 b	0.221 ± 0.002 b	0.163 ± 0.001 c	0.326 ± 0.007 a	0.253 ± 0.003 c	0.278 ± 0.004 b	0.248 ± 0.002 c
R2	0.982	0.866	0.894	0.771	0.982	0.914	0.897	0.924
Bingham								
τ0 B (Pa)	2.81 ± 0.086 d	3.60 ± 0.314 c	4.10 ± 0.152 b	6.17 ± 0.189 a	2.81 ± 0.086 b	1.84 ± 0.113 d	6.80 ± 0.157 a	2.1 ± 0.113 c
ηB (Pa s)	0.212 ± 0.002 a	0.171 ± 0.001 c	0.171 ± 0.002 c	0.181 ± 0.001 b	0.212 ± 0.002 a	0.114 ± 0.002 c	0.212 ± 0.003 a	0.124 ± 0.001 b
R2	0.925	0.951	0.952	0.884	0.925	0.961	0.901	0.955
Herschel-Bulkley								
kH (Pa·sn)	1.05 ± 0.010 d	3.22 ± 0.032 c	4.18 ± 0.018 b	5.30 ± 0.024 a	1.05 ± 0.010 d	1.15 ± 0.011 c	2.89 ± 0.033 a	1.71 ± 0.020 b
nH	0.761 ± 0.003 a	0.162 ± 0.002 c	0.491 ± 0.003 b	0.491 ± 0.002 b	0.761 ± 0.003 a	0.342 ± 0.001 c	0.421 ± 0.002 b	0.336 ± 0.001 d
R2	0.988	0.970	0.960	0.888	0.988	0.961	0.902	0.965
Casson								
τ0 c (Pa)	1.28 ± 0.015 d	1.64 ± 0.028 c	1.74 ± 0.041 b	2.24 ± 0.025 a	1.28 ± 0.015 b	1.13 ± 0.012 c	2.15 ± 0.010 a	1.22 ± 0.018 bc
ηc(Pa s)	0.360 ± 0.005 a	0.273 ± 0.004 b	0.272 ± 0.001 b	0.243 ± 0.001 c	0.360 ± 0.005 a	0.239 ± 0.004 d	0.321 ± 0.003 b	0.246 ± 0.002 c
R2	0.970	0.958	0.927	0.808	0.970	0.954	0.881	0.949

1, 2, and 3 as subscripts indicate the highest, moderate, and lowest CaCl_2_ concentrations (2, 5, and 10 mM) and NaCl (10, 50, and 100 mM), respectively; a–d: means followed by the same lower case in the same row are not significantly different (*p* > 0.05).

**Table 2:** Frequency dependence of elastic and viscous modulus of CSM–Blg complexes at 25 °C.

Treatments	*k′*	*n′*	R^2^	*k″*	*n″*	R^2^	*K″/k′*	*α*
CaCl_2_ (mM)								
0	2.66 ± 0.03 ^d^	0.140 ± 0.003 ^a^	0.956	0.77 ± 0.003 ^c^	0.292 ± 0.002 ^a^	0.991	0.290 ± 0.002 ^a^	0.631 ± 0.005 ^b^
2	3.15 ± 0.01 ^c^	0.125 ± 0.001 ^b^	0.973	0.82 ± 0.002 ^c^	0.278 ± 0.002 ^b^	0.992	0.266 ± 0.001 ^c^	0.659 ± 0.004 ^a^
5	3.51 ± 0.02 ^b^	0.112 ± 0.001 ^c^	0.950	0.98 ± 0.004 ^b^	0.212 ± 0.001 ^c^	0.980	0.280 ± 0.002 ^b^	0.516 ± 0.006 ^d^
10	4.35 ± 0.04 ^a^	0.096 ± 0.002 ^d^	0.991	1.23 ± 0.08 ^a^	0.189 ± 0.001 ^d^	0.983	0.283 ± 0.003 ^b^	0.597 ± 0.003 ^c^
NaCl (mM)								
0	2.66 ± 0.03 ^a^	0.140 ± 0.003 ^b^	0.956	0.77 ± 0.003 ^b^	0.292 ± 0.002 ^b^	0.991	0.290 ± 0.002 ^d^	0.631 ± 0.005 ^c^
10	1.37 ± 0.01 ^c^	0.134 ± 0.001 ^c^	0.987	0.70 ± 0.001 ^d^	0.311 ± 0.003 ^a^	0.987	0.513 ± 0.001 ^b^	1.25 ± 0.004 ^a^
50	1.82 ± 0.01 ^b^	0.148 ± 0.003 ^a^	0.985	0.84 ± 0.001 ^a^	0.148 ± 0.002 ^c^	0.954	0.464 ± 0.001 ^c^	0.452 ± 0.002 ^d^
100	0.880 ± 0.002 ^d^	0.105 ± 0.002 ^d^	0.993	0.73 ± 0.002 ^c^	0.137 ± 0.001 ^d^	0.948	0.821 ± 0.002 ^a^	1.06 ± 0.002 ^b^

a–d: Means followed by the same lower case in the same column are not significantly different (*p* > 0.05); the reported values are means (n = 3) ± SD.

**Table 3:** Intrinsic viscosity (dL/g) of CSM–Blg with the addition of salts at 25 °C.

Sample	Huggins	Kramer	Tanglertpaibul & Rao	Higiro 1	*R_coil_* (nm)	*V_coil_* (nm^3^)
CSM–Blg	R^2^ = 0.91 *η* = 0.97 ± 0.008	R^2^ = 0.88 *η* = 0.72 ± 0.004	R^2^ = 0.97 *η* = 0.42 ± 0.003	R^2^ = 0.98 *η* = 0.46 ± 0.003 ^a^	8.50 ± 1.00 ^a^	2662.0 ± 74.2 ^a^
CSM–Blg–Ca_1_	R^2^ = 0.89 *η* = 2.11 ± 0.21	R^2^ = 0.91 *η* = 1.80 ± 0.21	R^2^ = 0.95 *η* = 0.58 ± 0.003	R^2^ = 0.96 *η* = 0.32 ± 002 ^b^	7.76 ± 0.51 a^b^	1956.2 ± 58.7 ^b^
CSM–Blg–Ca_2_	R^2^ = 0.91 *η* = 1.80 ± 0.15	R^2^ = 0.93 *η* = 1.62 ± 0.14	R^2^ = 0.97 *η* = 0.41 ± 0.003	R^2^ = 0.98 *η* = 0.25 ± 002 ^d^	7.15 ± 0.34 ^a^	1530.3 ± 35.6 ^d^
CSM–Blg–Ca_3_	R^2^ = 0.92 *η* = 2.60 ± 0.30	R^2^ = 0.93 *η* = 2.27 ± 0.36	R^2^ = 0.94 *η* = 0.44 ± 0.004	R^2^ = 0.95 *η* = 0.28 ± 0.001 ^c^	7.33 ± 0.28 ^ab^	1648.2 ± 41.4 ^c^
CSM–Blg–Na_1_	R^2^ = 0.87 *η* = 1.76 ± 0.24	R^2^= 0.90 *η* = 1.60 ± 0.11	R^2^ = 0.93 *η* = 0.31 ± 0.004	R^2^ = 0.98 *η* = 0.20 ± 002 ^c^	6.64 ± 0.41 ^b^	1225.6 ± 24.9 ^c^
CSM–Blg–Na_2_	R^2^= 0.74 *η* = 1.18 ± 0.13	R^2^ = 0.80 *η* = 1.25 ± 0.08	R^2^ = 0.94 *η* = 0.40 ± 0.004	R^2^ = 0.95 *η* = 0.22 ± 0.001 ^b^	6.85 ± 0.21 ^b^	1345.2 ± 20.1 ^b^
CSM–Blg–Na_3_	R^2^ = 0.92 *η* = 1.36 ± 0.11	R^2^ = 0.93 *η* = 1.56 ± 0.13	R^2^ = 0.98 *η* = 0.35 ± 0.004	R^2^ = 0.98 *η* = 0.18 ± 0.001 ^d^	6.41 ± 0.16 ^b^	1102.2 ± 14.8 ^d^

1, 2, and 3 as subscripts indicate the highest, moderate, and lowest CaCl_2_ concentrations (2, 5, and 10 mM) and NaCl (10, 50, and 100 mM), respectively; a–d: means followed by the same lower case in the same row are not significantly different (*p* > 0.05).

## Data Availability

Data is contained within the article.
